# Gaps and complex structurally variant loci in phased genome assemblies

**DOI:** 10.1101/gr.277334.122

**Published:** 2023-04

**Authors:** David Porubsky, Mitchell R. Vollger, William T. Harvey, Allison N. Rozanski, Peter Ebert, Glenn Hickey, Patrick Hasenfeld, Ashley D. Sanders, Catherine Stober, Human Pangenome Reference Consortium, Jan O. Korbel, Benedict Paten, Tobias Marschall, Evan E. Eichler

**Affiliations:** 1 Department of Genome Sciences, University of Washington School of Medicine, Seattle, Washington 98195, USA;; 2 Institute for Medical Biometry and Bioinformatics, Medical Faculty, Heinrich Heine University, 40225 Düsseldorf, Germany;; 3 Center for Digital Medicine, Heinrich Heine University, 40225 Düsseldorf, Germany;; 4 UC Santa Cruz Genomics Institute, University of California Santa Cruz, Santa Cruz, California 95064, USA;; 5 European Molecular Biology Laboratory (EMBL), Genome Biology Unit, 69117 Heidelberg, Germany;; 6 Berlin Institute for Medical Systems Biology, Max Delbrück Center for Molecular Medicine in the Helmholtz Association, 10115 Berlin, Germany;; 7 Berlin Institute of Health (BIH), 10178 Berlin, Germany;; 8 Charité-Universitätsmedizin, 10117 Berlin, Germany;; 9 European Molecular Biology Laboratory, European Bioinformatics Institute, Wellcome Genome Campus, Hinxton, Cambridge, CB10 1SD, United Kingdom;; 10 Howard Hughes Medical Institute, University of Washington, Seattle, Washington 98195, USA;; 12 Division of Oncology, Department of Internal Medicine, Washington University School of Medicine, St. Louis, MO 63110, USA;; 13 McDonnell Genome Institute, Washington University School of Medicine, St. Louis, MO 63108, USA;; 14 UC Santa Cruz Genomics Institute, University of California, Santa Cruz, Santa Cruz, CA 95064, USA;; 15 Google LLC, Mountain View, CA 94043, USA;; 16 Department of Genome Sciences, University of Washington School of Medicine, Seattle, WA 98195, USA;; 17 European Molecular Biology Laboratory, European Bioinformatics Institute, Wellcome Genome Campus, Hinxton, Cambridge CB10 1SD, UK;; 18 Department of Human Genetics, McGill University, Montreal, Québec H3A 0C7, Canada;; 19 Canadian Center for Computational Genomics, McGill University, Montreal, Québec H3A 0G1, Canada;; 20 Institute for the Advanced Study of Human Biology (WPI-ASHBi), Kyoto University, Kyoto 606-8501, Japan;; 21 Institute of Genetics and Biophysics, National Research Council, Naples 80111, Italy;; 22 Department of Quantitative and Computational Biology, University of Southern California, Los Angeles, CA 90089, USA;; 23 Department of Data Sciences, Dana-Farber Cancer Institute, Boston, MA 02215, USA;; 24 Department of Biomedical Informatics, Harvard Medical School, Boston, MA 02215, USA;; 25 Department of Genetics, Genomics and Informatics, University of Tennessee Health Science Center, Memphis, TN 38163, USA;; 26 Arizona State University, Barrett and O'Connor Washington Center, Washington, DC 20006, USA;; 27 Department of Ecology and Evolutionary Biology, University of California, Santa Cruz, Santa Cruz, CA 95064, USA;; 28 Institute for Medical Biometry and Bioinformatics, Medical Faculty, Heinrich Heine University Düsseldorf, 40225 Düsseldorf, Germany;; 29 Center for Digital Medicine, Heinrich Heine University Düsseldorf, 40225 Düsseldorf, Germany;; 30 Core Unit Bioinformatics, Medical Faculty, Heinrich Heine University Düsseldorf, 40225 Düsseldorf, Germany;; 31 Howard Hughes Medical Institute, Chevy Chase, MD 20815, USA;; 32 Vertebrate Genome Laboratory, The Rockefeller University, New York, NY 10065, USA;; 33 National Institutes of Health (NIH)–National Human Genome Research Institute, Bethesda, MD 20892, USA;; 34 Department of Genetics, Washington University School of Medicine, St. Louis, MO 63110, USA;; 35 Center for Computational and Genomic Medicine, The Children's Hospital of Philadelphia, Philadelphia, PA 19104, USA;; 36 Novo Nordisk Foundation Center for Biosustainability, Technical University of Denmark, Copenhagen DK-2200, Denmark;; 37 Institute for Society and Genetics, College of Letters and Science, University of California, Los Angeles, Los Angeles, CA 90095, USA;; 38 Institute for Precision Health, David Geffen School of Medicine, University of California, Los Angeles, Los Angeles, CA 90095, USA;; 39 Division of General Internal Medicine and Health Services Research, David Geffen School of Medicine, University of California, Los Angeles, Los Angeles, CA 90095, USA;; 40 Department of Biomolecular Engineering, University of California, Santa Cruz, Santa Cruz, CA 95064, USA;; 41 Dovetail Genomics, Scotts Valley, CA 95066, USA;; 42 Quantitative Life Sciences, McGill University, Montreal, Québec H3A 0C7, Canada;; 43 Genomics Research Centre, Human Technopole, Milan 20157, Italy;; 44 Department of Genetics, Yale University School of Medicine, New Haven, CT 06510, USA;; 45 Center for Genomic Health, Yale University School of Medicine, New Haven, CT 06510, USA;; 46 Quantitative Biology Center (QBiC), University of Tübingen, 72076 Tübingen, Germany;; 47 Biomedical Data Science, Department of Computer Science, University of Tübingen, 72076 Tübingen, Germany;; 48 Tree of Life, Wellcome Sanger Institute, Hinxton, Cambridge CB10 1SA, UK;; 49 Northeastern University, Boston, MA 02115, USA;; 50 Laboratory of Neurogenetics of Language, The Rockefeller University, New York, NY 10065, USA;; 51 Division of Oncology, Department of Medicine, Stanford University School of Medicine, Stanford, CA 94305, USA;; 52 Institute for Genomic Health, Icahn School of Medicine at Mount Sinai, New York, NY 10029, USA;; 53 Program in Bioethics and Institute for Human Genetics, University of California, San Francisco, San Francisco, CA 94143, USA;; 54 European Molecular Biology Laboratory, Genome Biology Unit, 69117 Heidelberg, Germany;; 55 Genome Informatics Section, Computational and Statistical Genomics Branch, National Human Genome Research Institute, National Institutes of Health, Bethesda, MD 20892, USA;; 56 Division of Biology and Biomedical Sciences, Washington University School of Medicine, St. Louis, MO 63110, USA;; 57 Computer Sciences Department, Barcelona Supercomputing Center, 08034 Barcelona, Spain;; 58 Departament d'Arquitectura de Computadors i Sistemes Operatius, Universitat Autònoma de Barcelona, 08193 Barcelona, Spain;; 59 Material Measurement Laboratory, National Institute of Standards and Technology, Gaithersburg, MD 20877, USA;; 60 Coriell Institute for Medical Research, Camden, NJ 08103, USA;; 61 Department of Computer Science, University of Pisa, Pisa 56127, Italy;; 62 Department of Public Health Sciences, University of California, Davis, Davis, CA 95616, USA;; 63 Department of Biomedical Engineering, Johns Hopkins University, Baltimore, MD 21218, USA;; 64 Department of Ecology and Evolutionary Biology, University of California, Santa Cruz, Santa Cruz, CA 95064, USA;; 65 Berlin Institute for Medical Systems Biology, Max Delbrück Center for Molecular Medicine in the Helmholtz Association, 10115 Berlin, Germany;; 66 National Center for Biotechnology Information, National Library of Medicine, National Institutes of Health, Bethesda, MD 20894, USA;; 67 Center for Health Data Science, University of Copenhagen, 2200 Copenhagen, Denmark;; 68 Al Jalila Genomics Center of Excellence, Al Jalila Children's Specialty Hospital, Dubai, UAE;; 69 Center for Genomic Discovery, Mohammed Bin Rashid University of Medicine and Health Sciences, Dubai, UAE;; 70 Division of Medical Genetics, University of Washington School of Medicine, Seattle, WA 98195, USA;; 71 Center for Computational Biology, Johns Hopkins University, Baltimore, MD 21218, USA;

## Abstract

There has been tremendous progress in phased genome assembly production by combining long-read data with parental information or linked-read data. Nevertheless, a typical phased genome assembly generated by trio-hifiasm still generates more than 140 gaps. We perform a detailed analysis of gaps, assembly breaks, and misorientations from 182 haploid assemblies obtained from a diversity panel of 77 unique human samples. Although trio-based approaches using HiFi are the current gold standard, chromosome-wide phasing accuracy is comparable when using Strand-seq instead of parental data. Importantly, the majority of assembly gaps cluster near the largest and most identical repeats (including segmental duplications [35.4%], satellite DNA [22.3%], or regions enriched in GA/AT-rich DNA [27.4%]). Consequently, 1513 protein-coding genes overlap assembly gaps in at least one haplotype, and 231 are recurrently disrupted or missing from five or more haplotypes. Furthermore, we estimate that 6–7 Mbp of DNA are misorientated per haplotype irrespective of whether trio-free or trio-based approaches are used. Of these misorientations, 81% correspond to bona fide large inversion polymorphisms in the human species, most of which are flanked by large segmental duplications. We also identify large-scale alignment discontinuities consistent with 11.9 Mbp of deletions and 161.4 Mbp of insertions per haploid genome. Although 99% of this variation corresponds to satellite DNA, we identify 230 regions of euchromatic DNA with frequent expansions and contractions, nearly half of which overlap with 197 protein-coding genes. Such variable and incompletely assembled regions are important targets for future algorithmic development and pangenome representation.

The past two years have witnessed tremendous progress with respect to advances in sequencing technology (Lu et al. 2016; Vollger et al. 2019; Wenger et al. 2019), as well as numerous assembly strategies that now make it possible to phase and assemble >95% of the content of a diploid genome (Logsdon et al. 2021; Jarvis et al. 2022). Because of these developments, genome assemblies have changed in two significant ways. We no longer consider collapsed 3-Gbp genome assemblies as state of the art (i.e., one representation of an individual where both haplotypes are merged) but instead consider two genomes for every diploid genome assembled (i.e., 6 Gbp vs. 3 Gbp) where parental haplotypes are phased and fully resolved. Second and in part because of the first, the number of gaps being produced has been reduced from thousands to only a few hundred. As a result, there have been a series of efforts to generate more complete and phased human genome assemblies using long-read sequencing platforms, including the Human Genome Structural Variation Consortium (HGSVC) and the Human Pangenome Reference Consortium (HPRC) (Ebert et al. 2021; Liao et al. 2023). Efforts such as these have generated data with different sequence technologies and applied different algorithms and strategies to generate multiple phased human genomes, including some that now rival the contiguity and accuracy of the current human genome reference (GRCh38).

In particular, the development of Pacific Biosciences (PacBio) high-fidelity (HiFi) reads, based on circular consensus sequencing (CCS) technology, provides ∼20-kbp sequencing reads that compete with short reads with respect to their accuracy (QV > 30), whereas the Oxford Nanopore Technologies (ONT) platform now can routinely generate sequencing reads in excess of 100 kbp (so-called ultralong [UL] sequencing reads) (Nurk et al. 2020; Shafin et al. 2020; Logsdon et al. 2021). The use of parent–child trio (trio-hifiasm) Illumina whole-genome sequencing (WGS) data in conjunction with CCS data provides the greatest power to phase a genome into its constituent paternal and maternal haplotypes. In the absence of parental data, however, methods have been developed (PGAS and HiC-hifiasm) using linked-read data, such as Strand-seq (Porubsky et al. 2021) or Hi-C (Garg et al. 2021; Cheng et al. 2022), that can phase genomes at the local and chromosomal level.

The challenge that remains is routine telomere-to-telomere (T2T) assembly of human genomes such that the full genetic diversity of species can be understood. Assembly gaps are, unfortunately, still an integral feature of every de novo diploid genome assembly. This status quo will remain until the sequencing technology and assembly algorithms evolve so that each homologous chromosome of any genome can be routinely assembled T2T in an automated fashion. Key to this aspirational goal is understanding why gaps persist, which in turn requires a detailed analysis of gap size, frequency, genomic location, and the sequence properties that define these regions. With the completion and annotation of the first T2T genome (Nurk et al. 2022), we are in a position to characterize the properties of the gaps that remain when diploid human genomes are routinely sequenced. We focus on a detailed characterization of these remaining gaps in an effort to understand their origin, biology, and the relative importance of getting these through the last impasses to T2T assembly. We focus on human diploid genomes because resolution of the gaps will improve discovery of both disease-related variation as well as genetic changes important for the evolution and adaptation of our species.

## Results

We investigated the gaps and contig breaks in a total of 182 haploid assemblies obtained from a diversity panel of 77 unique human samples sequenced with long-read technology. The underlying long-read data and assemblies were generated by two consortia over the past two years, HGSVC (88 assemblies) and HPRC (94 assemblies), using different long-read sequencing platforms as well as assembly strategies. The HGSVC used two different long-read sequencing technologies, continuous long-read (CLR; 60 assemblies) sequencing (Ebert et al. 2021) and CCS (or HiFi sequencing, 28 assemblies) with an additional eight samples shared between HGSCV and HPRC used only for validation purposes. CCS and CLR data from HGSVC were assembled using a trio-free assembly pipeline, called PGAS (Ebert et al. 2021; Porubsky et al. 2021; Ebler et al. 2022) using both the Peregrine (Chin and Khalak 2019) (PGASv12) and the hifiasm (Cheng et al. 2021) (PGASv13) assemblers for CCS and the Flye assembler ((Kolmogorov et al. 2019) for CLR data. The HPRC effort, which began more than a year later, focused exclusively on CCS data (n = 94) generated from diploid samples assembled using trio-based hifiasm (Cheng et al. 2021). Here, parent–child data were directly used to aid assembly phasing of all HPRC samples (Wang et al. 2022; Liao et al. 2023), allowing for both platform and methodology comparisons (Supplemental Table S1; Supplemental Fig. S1A).

### Evaluation metrics and gap definitions

**Figure 1.**
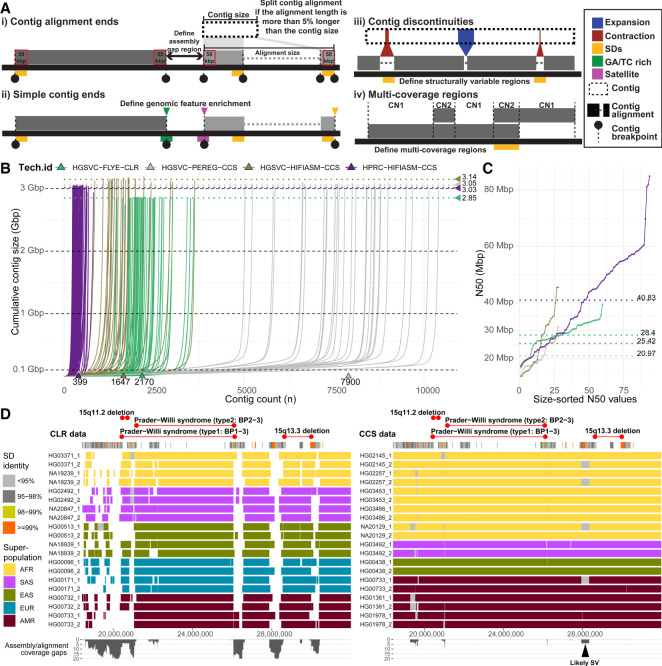
Comparison and evaluation of phased assemblies. (*A*) Assembly metrics evaluated in this study. (*i*) Contig alignment ends are defined as terminal contig alignments such that the total alignment size does not exceed the actual contig size by >5%. When this requirement is not met, multiple contig end alignments will be reported. (*ii*) Simple contig ends are defined as the first and last alignments of each contig to the reference (T2T-CHM13 v1.1) with at least 25 kbp aligned. (*iii*) Contig discontinuities are defined as alignment gaps between subsequent pieces of a single contig <1 Mbp. (*iv*) Detection of regions with coverage more than 1n as is expected for a haploid genome. (*B*) A cumulative contig size distribution colored by assembly technology. Each line represents a single haploid assembly (HGSVC-FLYE-CLR, n = 60; HGSVC-PEREG-CCS, n = 28; HGSVC-HIFIASM-CCS, n = 28; HPRC-HIFIASM-CCS, n = 94). Median total assembly length per assembly technology is highlighted as horizontal dotted lines. (*C*) Contig N50 values colored by assembly technology as in *B*. Each dot represents a single haploid assembly. Median N50 value per assembly technology is highlighted as horizontal dotted lines. (*D*) Track definition from *top* to *bottom*: Regions corresponding to known genomic disorders between 15q11.2–15q13.3. *Below* is the annotation of SDs in this region colored by sequence identity. Main track shows the visualization of contig alignments for 10 random samples from trio-free CLR assemblies (*left*) in comparison to trio-based HPRC assemblies (*right*). Contig alignments are colored by sample superpopulation (AFR, African; SAS, Southeast Asian; EAS, East Asian; EUR, European; AMR, American). White spaces between contig alignments represent boundaries between subsequent contig. Spaces filled with gray color represent unaligned portions of a single contig with respect to the reference (T2T-CHM13) and likely represent a structural variation (black arrowhead). The last track summarizes the extent of assembly gaps (between contigs; white space) and contig gaps (within contigs; gray rectangles) as coverage plot.

In this study, we set out to evaluate assembly quality and completeness using four metrics (Methods). We start with defining regions between subsequent contigs mapped to the T2T-CHM13 human genome reference. These are defined based on reliable “contig end alignments” (≥50 kbp at the contig edges) mapped in agreement with an expected contig length. Contig end alignments were used to localize regions (assembly gaps) in between subsequent contigs (Fig. 1A, i). Second, we define “simple contig ends” as terminal contig positions with respect to the reference genome. Simple contig ends were used for enrichment analysis of various genomic features near terminal contig alignment positions (Fig. 1A, ii). To evaluate structural differences between assemblies, we set to document all regions that break contig alignments, referred to here as “contig alignments discontinuities.” We focus on discontinuities that create internal gaps within contig alignments <1 Mbp in length to document regions of putative structural differences that cannot be readily aligned to a single reference (Fig. 1A, iii). Lastly, we turn our attention to regions with a higher coverage than expected in a haploid genome (multicoverage regions) caused by two or more overlapping contig alignments. Such regions point to positions of either true structural differences or genome assembly artifacts (Fig. 1A, iv).

### Platform and assembly method comparisons

We initially compared assembly statistics between different sequencing technologies and assembly algorithms to determine what combination provides the most continuous and complete assembly. The most fragmented assemblies were obtained using a combination of the trio-free PGAS pipeline and the Peregrine assembler with a median contig count of 7900 per assembly (Ebert et al. 2021). Improved contiguity was achieved by combination of the PGAS pipeline and CLR data assembled by Flye (median contigs, 2170) and CCS data assembled by hifiasm (median contigs, 1647) (Ebert et al. 2021; Ebler et al. 2022). The most continuous assemblies were obtained using the trio-based hifiasm assembly, resulting in an order of magnitude fewer gaps (e.g., 399 median contigs per assembly) (Fig. 1B). The least complete assemblies resulted from a combination of PGAS and CLR data (median size, 2.85 Gbp). This is expected because higher error rates of CLR in comparison to CCS data prevent them from assembling highly identical segmental duplications (SDs) in the human genome. Assemblies using CCS data provide comparable assembly completeness (median size, ∼3.05 Gbp) with a slightly higher median assembly size for the trio-free PGAS pipeline combined with hifiasm (median size, 3.14 Gbp) (Fig. 1B). Lastly, the assembly contiguity was evaluated as a function of contig N50, and again, we conclude that trio-based assembly (N50, 40.83 Mbp) outperforms those assembled in trio-free settings (Fig. 1C). Because of suboptimal performance, we excluded Peregrine assemblies from subsequent analyses.

Consistent with a recent study (Jarvis et al. 2022), trio-based assemblies contain the least number of gaps between contig alignment ends (median, 141) followed by PGAS-hifiasm with about double that amount (median, 320) and PGAS-Flye (median, 392) (Supplemental Fig. S1B). Based on projections to the T2T-CHM13 reference, the number of missing base pairs follows a similar trend, with trio-based assemblies having the least number of bases within gaps between defined contig alignment ends (median, 78.4 Mbp) followed by PGAS-hifiasm (median, 126.7 Mbp) and PGAS-Flye (median, 244.8 Mbp) (Supplemental Fig. S1C), although there are outliers (Supplemental Fig. S2). CCS-based assemblies are generally superior to those produced from CLR because highly identical SDs, including disease-relevant regions such as Prader–Willi, are largely absent from CLR-based assemblies (Fig. 1D, white gaps). As a result, ∼59.9 Mbp is missing in CLR assemblies in contrast to only ∼690 kbp in CCS-based assemblies, allowing us to begin to assess SD-associated copy number variation and structural variation (Fig. 1D, gray gaps). Given these observations, we exclude CLR-based assemblies from subsequent analysis and focus exclusively on CCS-hifiasm assemblies.

### Parent–child trio-based versus trio-free assemblies

**Figure 2.**
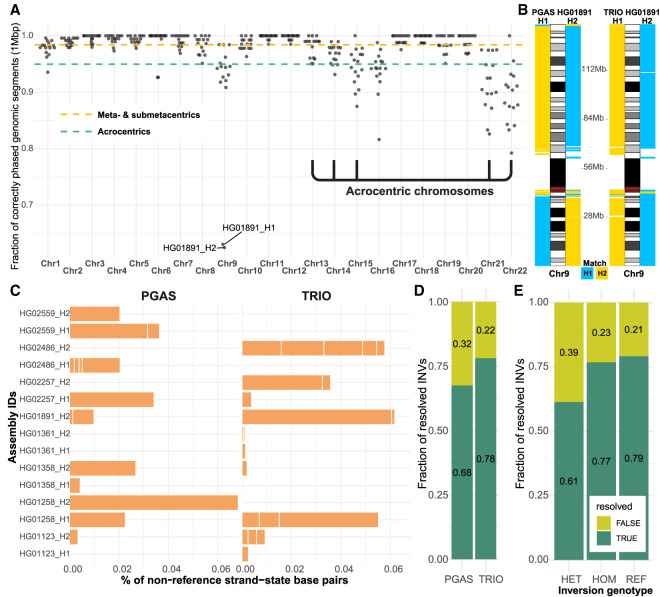
Phasing accuracy and inversion analysis of trio-based and trio-free assemblies. (*A*) Phasing accuracy of PGAS (trio-free) assemblies with respect to trio-based phasing. (*B*) Haplotype assignment of 1-Mbp-sized blocks (*left* from ideogram, H1; *right* from ideogram, H2) to either haplotype 1 or 2 (blue, H1; yellow, H2) using single-nucleotide polymorphisms phased using trio information (1000 Genomes Project panel) with respect to the reference (GRCh38). (*C*) A barplot reporting the percentage of base pairs in an opposite (reverse) orientation in contrast to the expected (direct) orientation based on Strand-seq analysis of assembly directionality, shown separately for trio-free (PGAS, n = 15; *left*) and trio-based (TRIO, n = 23; *right*) assemblies. (*D*) Fraction of tested inversion sites that are fully informative (TRUE; dark green). (*E*) Fraction of tested inversion sites that are fully informative (TRUE; dark green) as a function of inversion genotype. (HET) Heterozygous, (HOM) homozygous inverted, (REF) homozygous reference.

We compared in more detail eight human genomes for which both long-range linked reads (Strand-seq) and parental data (Illumina WGS) were available from the same individuals. Using the same underlying long-read input data (CCS), we specifically performed a head-to-head comparison of trio-based (TRIO; using parental Illumina WGS for phasing) and trio-free (PGAS; using Strand-seq for phasing) assemblies. We find that assemblies generated in the absence of parental data (trio-free) have about twice as many contigs and a decreased contig N50 by ∼10 Mbp (Supplemental Fig. S3), likely because the underlying assembly algorithm reuses paths as opposed to generating a primary and alternate in the absence of parental data. We next evaluated phasing accuracy of trio-free assemblies using the genomes phased by parental data as the truth set (Methods). For the metacentric and submetacentric chromosomes, we observe a high accuracy of phased 1-Mbp segments, achieving 98% concordance with trio-based phasing. With acrocentric chromosomes, this accuracy drops to 94% (Fig. 2A; Supplemental Fig. S4). The majority of incorrectly assigned 1-Mbp segments (>75%) map within centromeric satellite repeats, most likely owing to the lower density of uniquely mapped single-nucleotide variants (SNVs) (Supplemental Fig. S5). There was only one sample (HG01891) with large-scale switch errors on a short arm of Chromosome 9 (∼42 Mbp) and one at the very end of Chromosome 9 (∼1 Mbp) (Fig. 2B). The data show that trio-free assemblies provide comparable phasing accuracy and completeness and are a viable option for phased genome assembly for samples in which parental data are not easily available or are cost prohibitive.

Strand-seq also preserves directionality of single-stranded DNA and thus is also able to unambiguously define misoriented regions of the genome. Such misorientations will appear as unresolved homozygous inversions based on Strand-seq reads mapping from the original genome sample (Methods). We detected comparable numbers of unresolved homozygous inverted regions in trio-based (n = 23) and trio-free (n = 15) assemblies, respectively (Supplemental Table S2), resulting in 6.8 Mbp (0.23%) and 7.3 Mbp (0.25%) of misoriented base pairs per assembly (Fig. 2C). The majority (31/38, >81%) of these misorientations overlap with previously defined true inversion polymorphisms in the human genome (Porubsky et al. 2022), six of which were unresolved in both trio-based and trio-free assemblies (Supplemental Fig. S6A) and, as expected, are flanked by large tracts of SDs (Supplemental Fig. S6B). Some of these span genomic disorder critical regions where recurrent de novo copy number variants (CNVs) associate with neurodevelopmental delay, such as the 16p11.2–p12.2 microdeletion and microduplication (Supplemental Fig. S7).

We more systematically evaluated the potential of both assembly approaches to resolve known large (≥100 kbp, n = 20) inversions considering both heterozygous as well as homozygous sites (Methods). Trio-based assemblies resolve 78% of inversion polymorphisms, whereas trio-free assemblies resolve 68% (Fig. 2D). Trio-based approaches generally more accurately represent more inverted base pairs (64%) compared with the trio-free approach (48%) by virtue of the fact they often assemble one end of an inversion polymorphism (Supplemental Fig. S8A,B). It is notable that nearly a quarter of all large inversion polymorphisms are not accurately represented in existing trio-based genome assemblies, with heterozygous inversions being the most difficult to fully resolve (Fig. 2E; Supplemental Fig. S8C). All sites (n = 14) that are unresolved two or more times in trio-based and trio-free assemblies are flanked by large (>40 kbp; median, 228.2 kbp) highly identical SDs (median, 99.4%). The availability of Strand-seq data provides a valuable orthogonal method for detection of such errors in the assembly, which in turn can guide targeted reassemblies of such regions using UL ONT reads.

### Sequence properties of the gaps

**Figure 3.**
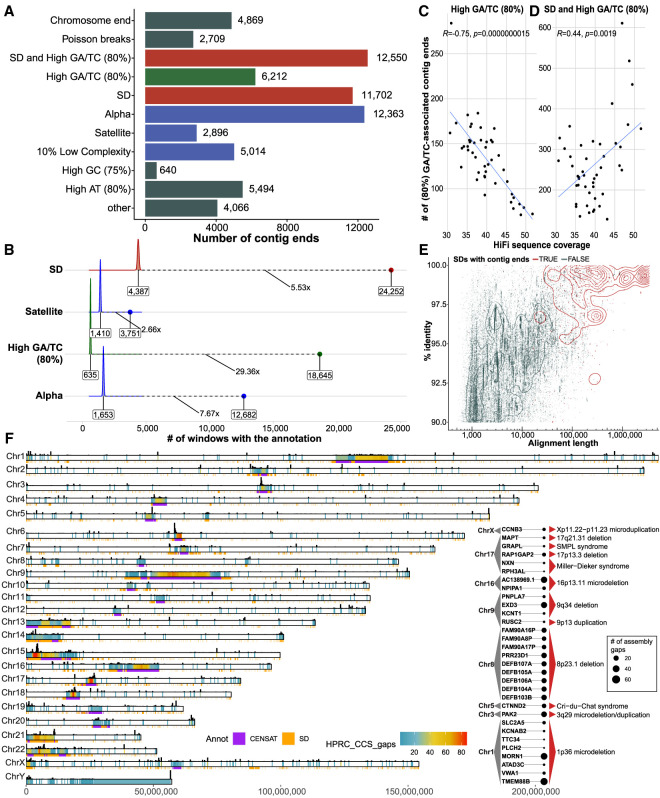
Sequence properties at defined contig ends. (*A*) The number of simple contig ends that are within or near (at most 10 kbp) a particular sequence annotation. Annotations are nonredundant and are prioritized in the order shown; for example, if a contig end is near the end of a chromosome and in an SD, it will only be annotated as a chromosome end. Note that chromosome ends are contig ends within the last 100 kbp of contigs. Poisson ends are contig ends that happen in only one haplotype (nonrecurrent and therefore likely to be random). SD and high GA/TC mean that the end is within 10 kbp of an SD and within 10 kbp of a 1-kbp window with at least 80% GA/TC content. (*B*) The fold enrichment in the number of contigs ends within 10 kbp of a sequence annotation compared with a distribution of randomly placed contig end simulations (10,000 permutations). Shown in text is the median of the random distribution (*left*), the fold enrichment (*middle*), and the observed value (*right*). In this analysis contig ends may exist in multiple categories; for example, if a contig end is near both an SD and a satellite sequence, it will appear in both simulations. (*C*) The effect of HiFi coverage on number of GA/TC breaks is negatively correlated when considered independently; however, when combined with SDs, the trend is inverted, as shown in *D*. (*E*) All SDs in T2T-CHM13 displayed by their length and percentage of identity (blue) versus the SDs that intersect contig ends (red). (*F*) Genome-wide distribution of gaps defined in between contig alignment ends (Methods) across all HPRC assemblies (n = 94). Color range reflects the number of assembly gaps overlapping each other in any given genomic region. On the *top* of each chromosomal bar, there is a density of simple contig ends. The height of each bar reflects the number of simple contig ends counted in 200-kbp-long genomic bins. *Inset*: List of protein-coding genes (n = 31) overlapping assembly breaks and reported microdeletion and microduplication syndromes.

Because the HPRC-phased genome assemblies represent the current state of the art in terms of both accuracy, phasing, and contiguity (Figs. 1, 2), we focused on a more in-depth analysis of sequence content of gap regions by mapping all sequence contigs to the complete human reference (T2T-CHM13, v1.1) (Nurk et al. 2022). Among the 94 HPRC haplotype assemblies, we identified a total of 68,515 simple contig ends for an average of 729 per haplotype (median, 700) (Fig. 1C; Supplemental Table S3). Of these contig breaks, about two-thirds correspond to SDs (35.4%; [11,702 + 12,550]/68,515) or satellite DNA (22.3%; [2896 + 12,363]/68,515) (Fig. 3A). Because long tracts of GA repeats have been predicted to reduce the coverage of CCS data (Nurk et al. 2020), it is important to note that 27.4% ([6212 + 12,550]/68,515) of the gaps, including recurring gaps, within the assemblies correspond to regions where high GA/TC tracts are observed (1-kbp window with >80% GA/TC within 10 kbp). These GA/TC tracts show the most substantial (29.36-fold) (Fig. 3B) enrichment for gaps and, along with high AT content, account for ∼40% of the assembly breaks not associated with large repetitive sequences ([6212 + 5494]/[68,515 − 2896 − 12,363 − 11,702 − 12,550]). Controlling for sequence coverage, we estimate that nearly two-thirds of the GA/TC gaps can be remedied by simply increasing sequence coverage from approximately 30- to 50-fold (Fig. 3C). However, we also find long tracts of GA/TC repeats nonrandomly associated with regions of SDs (Fig. 3A). In such regions, increasing coverage has little effect on reducing the number of gaps and perhaps has even the opposite effect (Fig. 3D). We considered both the length and sequence identity of SDs and found that the longer and more identical an SD is, the more likely it was associated with a gap. Thus, the longest and most identical SDs are preferentially associated with gaps in the majority of analyzed assemblies (Fig. 3E; Supplemental Fig. S9).

Despite the differences in contig end definition, we found a high level of agreement between simple contig ends and assembly gap regions, with >85% of simple contig ends falling into assembly gaps and >99% of assembly gaps overlapping with simple contig ends (Fig. 3F; Supplemental Fig. S10). Assembly gaps are regions that are not completely assembled across HPRC assemblies. This is especially problematic when assessing human diversity among protein-coding genes. The whole set of assembly gaps (n = 14,662) from all HPRC assemblies overlaps a total of 1513 protein-coding genes (Supplemental Fig. S11) that fall within 894 nonredundant gap regions. There are 231 protein-coding genes that fall within regions broken in five or more HPRC assemblies (Supplemental Fig. S12; Supplemental Table S4), and 31 of these lie within regions of recurrent microdeletion and microduplication syndromes (Cooper et al. 2011; Coe et al. 2014). Among these, there are a number of biomedically relevant genes, such as *PAK2* affected by 3q29 microdeletion, *CTNND2* affected in Cri-du-Chat syndrome, or *MAPT* affected by 17q21.31 microdeletion (Fig. 3F, inset).

**Figure 4.**
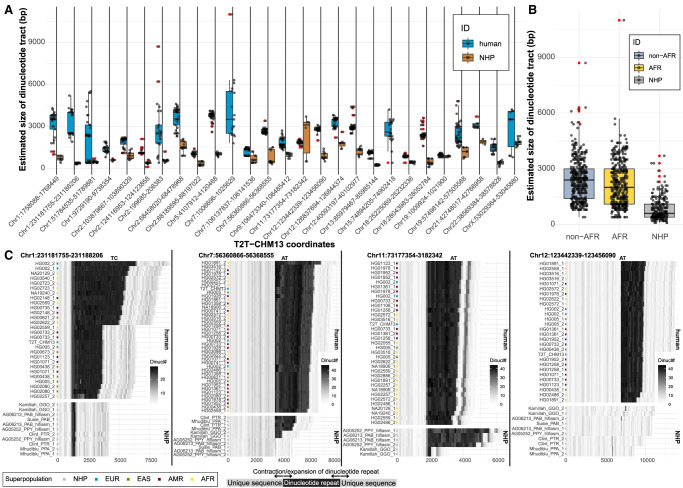
Sequence variation in low-complexity regions. (*A*) Size distribution comparison of dinucleotide tracts (*y*-axis) between human (blue) and nonhuman primates (NHPs; brown) for 27 selected regions (Methods). Outliers are highlighted as red dots. (*B*) A summary of size distribution of dinucleotide tracts (*y*-axis) between human samples of African (AFR; yellow) and non-African (non-AFR; light blue) origin and NHPs (gray) across all complete assemblies from 27 selected regions. (*C*) Difference in dinucleotide frequency (TC, AT) between humans and NHP in four genomic regions. Shades of gray color reflect the number of detected dinucleotides (defined at the *top* of each plot) in 100-bp-long DNA sequence chunks. Assembly names (*y*-axis) from NHP contain sample IDs and species-specific ID: (PTR) *Pan troglodytes*, (GGO) *Gorilla gorilla*, (PPA) *Pan paniscus*, (MMU) *Macaca mulatta*, (PAB) *Pongo abelii*, (PPY) *Pongo pygmaeus*. Numbers 1 and 2 represent parental homolog IDs of given sample assembly.

Overall, we define 592 nonredundant regions, outside of satellite DNA, with an assembly gap in five or more of the HPRC assemblies (Supplemental Fig. S13; Supplemental Table S5). Among the most recurrent gaps, there are 44 euchromatic regions that fail to resolve in half or more of the HPRC assemblies. Although a third of these are associated with SDs, 28 of these are dropouts associated with the presence of low-complexity DNA (Supplemental Table S6). In these 28 regions, we observe continuous tracts of dinucleotides (AT or GA/TC) ranging from ∼300–6500 kbp in the T2T-CHM13 reference (Supplemental Fig. S14); however, we noticed several such low-complexity tracts in regions associated with SDs (n = 16) (Supplemental Fig. S15). We further explored the extent of the variability in size of low-complexity regions between humans and nonhuman primates in assemblies that managed to span these regions (Methods). We catalog 27/44 regions with observable differences in size of dinucleotide tracts, with humans carrying longer dinucleotide tracts in all but one instance (Fig. 4A). Our analysis suggests that many of these regions appear to have expanded specifically in the human lineage, where they continue to show variability in size (Fig. 4B,C).

### Discontinuous alignments and large structural variants

**Figure 5.**
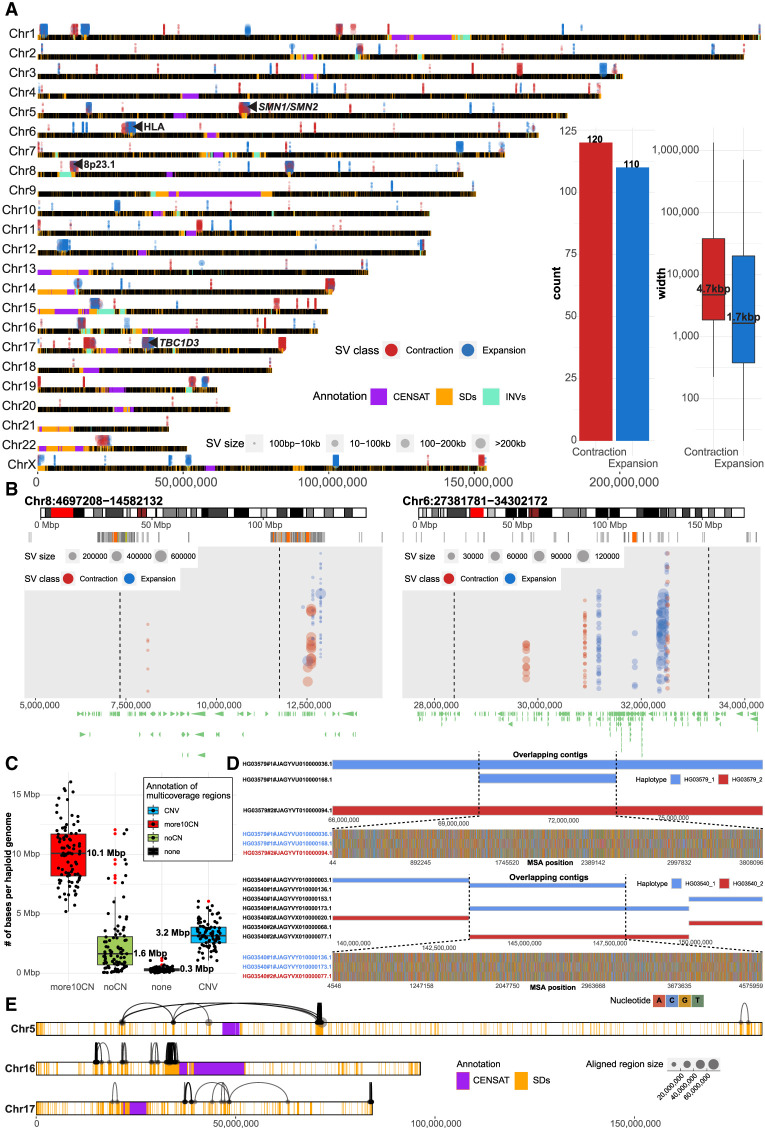
Tracking contig alignment discontinuities and multicoverage regions. (*A*) Genome-wide distribution of frequent (n = 230) contig alignment discontinuities (1 kbp to 1 Mbp in size). Each gap is represented in each separate assembly (HPRC, 94; HGSVC, 28) by a colored dot (blue, expansion [INS]; red, contraction [DEL]), and the size of each dot represents the size of the event in contig coordinates. A region is defined as an INS (blue) if there is a gap in a contig alignment (in reference T2T-CHM13, v1.1 coordinates) that is smaller than the sequence within a contig itself delineated by the *left* and *right* alignments flanking the gap. In contrast, a DEL (red) is defined as a gap in a contig alignment (in reference T2T-CHM13, v1.1 coordinates) that is larger than the sequence within a contig itself delineated by the *left* and *right* alignments around the gap. Putative expansions and contractions above the horizontal chromosomal lines were detected in HPRC assemblies, and those below the lines in HGSVC assemblies. Centromeric satellite regions are highlighted by gray rectangles and regions of segmental duplications (SDs) as orange rectangles on top of each chromosomal line (black). (*B*) Example regions (*left*, defensin locus, 8p23.1; *right*, HLA locus) with frequent expansions and contractions. Each region is highlighted as a red rectangle on chromosome-specific ideogram (*top* track). *Below*, there is an SD annotation for a given region represented as a set of rectangles colored by sequence identity. Expansions and contractions of each contig alignment with respect to the reference (T2T-CHM13, v1.1) are depicted as blue and red dots, respectively. The size of each dot represents the size of an event. (*C*) Assignment of total number base pairs covered by multiple contig alignments, in each haploid genome (n = 88), into four categories based on agreement with short-read-based CNV profiles (for detailed description of categories, see Methods). (*D*) Example regions in samples HG03579 and HG03540, where overlapping contigs associate with loss of heterozygosity. *Top* track shows contig alignments in a given region separately for haplotype 1 (blue; paternal) and haplotype 2 (red; maternal). Overlapping contig alignments are stacked on *top* of each other. The *bottom* track shows all variable positions detected in a multiple sequence alignment (MSA) over the region where contigs overlap (dashed lines). Here, one of the paternal contigs is nearly identical to a maternal contig at the region where contigs overlap. (*E*) Chromosomes 5, 16, and 17 are depicted as horizontal bars with the locations of SDs and centromeric regions highlighted as orange and purple rectangles, respectively. Contig alignment ends divided into multiple pieces are visualized as links between subsequent pieces of a single contig aligned to the reference (T2T-CHM13 v1.1). The length of the aligned pieces of a contig are defined by the size of each dot.

One of the advantages of the new assemblies of the human genome is that they are not guided by existing human references. Such de novo assemblies have the potential to identify large discontinuities corresponding to potential larger forms of genetic variation, including partially sequence-resolved CNVs. We searched specifically for contig alignment discontinuities (<1 Mbp) as identified by alignment to the complete human reference genome (T2T-CHM13, v1.1; Methods) (Fig. 1A). Across all 94 human haplotypes, we report a median 6.6% and 0.06% of unaligned bases per assembly within and outside of centromeric satellite DNA, respectively (Supplemental Fig. S16). Per haploid genome, we define a median number of 165 contractions and 262 expansions, which corresponds to ∼11.9 Mbp and 161.4 Mbp, respectively (Supplemental Fig. S17A,B). The vast majority of these bases (contractions, 10.9 Mbp; expansions, 159.8 Mbp) belong to centromeric satellite DNA, which are known to vary extensively in size and composition among human haplotypes and are often incompletely assembled (Supplemental Fig. S17C). Nevertheless, within euchromatic regions, we identified 230 regions that showed evidence of contraction (n = 120) or expansion (n = 110) in multiple human haplotypes (five or more) compared with the T2T-CHM13 reference (Fig. 5A; Supplemental Table S7). A large number of these regions overlap with SDs (∼40%, 93/230) and include biomedically relevant loci that are known to be structurally variable, such as 8p23.1, HLA, *SMN1/SMN2*, and *TBC1D3* (Fig. 5B; Supplemental Fig. S18; Vollger et al. 2022). Based on the read-depth analysis of Illumina WGS data, we confirm 41 of these regions: the majority of which correspond to copy number losses in their respective genomes (Methods) (Supplemental Fig. S19). We highlight a region on Chromosome 11 (Chr 11: 55,535,304–55,628,574, 11q12.1) where the contracted region (∼93 kbp) is associated with short inversion (∼4 kbp) that flips *OR4C6* into a direct orientation with respect to *OR4C11*, which likely promotes a microdeletion via nonallelic homologous recombination (NAHR) as this deletion is observed in association with an inverted haplotype (Supplemental Fig. S20).

In addition to the assembled sequence that does not readily map to the reference, we also cataloged regions where there are multiple contig mappings (more than one) instead of the expected haploid single copy (Fig. 1A, iv). Per haplotype, we observe ∼15.4 Mbp of euchromatic sequence with multiple contig mappings with respect to the reference (T2T-CHM13, v1.1). Although such multimapping regions likely represent CNV regions arising from SD, they may also result from ambiguous contig mappings or artifacts of the assembly process. Improved mapping and assembly algorithms will be required to understand the biological significance of these regions. To enrich for true CNVs, we searched for CNV regions that were also supported by read-depth analysis of short-read data (Methods). Indeed, we identified ∼3.2 Mbp predicted to be CNV (2–10 copies) and supported by short-read sequence data. An even greater fraction (∼10.1 Mbp) of multimapping regions show greater CNVs (more than 10 copies) based on short-read depth, although the true copy number is more difficult to determine as the majority (>95%) of these regions overlap with SDs by >90%.

Nevertheless, we identify ∼1.6 Mbp per haplotype of multimapping regions where we find no obvious CNV in short-read data (Fig. 5C). We note that a subset of these are large (≥500 kbp) and often (85/118) represent sequence contigs that are completely embedded within another larger contig in a single haplotype. We investigated eight of the longest such contigs in more detail (Methods). Comparison of heterozygous SNV patterns across these regions based on CCS data (DeepVariant calls) and phased assemblies (dipcalls) reveals conspicuous stretches of loss of heterozygosity over the region where the multimapping contigs overlap (Supplemental Fig. S21). Closer inspection reveals that the sequence variation between parental haplotypes is, however, not lost but rather present only in one contig, whereas the other contig is nearly identical to the other parental haplotype (Fig. 5D). Although the origin of such assembly artifacts is unclear, such overlapping contigs will likely pose challenges for SNV calling depending on which, if any, sequence contig is chosen.

We focused specifically on euchromatic regions where both long- and short-read data were in agreement regarding increased copy number variation (a fewer than 10 copy number increase with respect to the reference). We identified 255 nonredundant CNV regions that encompass 44.9 Mbp of the genome (Supplemental Table S8). Of these CNV regions, 87% (39.1 Mbp) correspond to SDs that are known to be copy number variable because of their propensity to undergo NAHR (Supplemental Fig. S22; Sharp et al. 2006; Sudmant et al. 2010, 2015). We find that genomes of African ancestry carry more CNV bases (∼3.5 Mbp) compared with other non-African populations (Supplemental Fig. S23) consistent with previous reports (Sudmant et al. 2015; Chaisson et al. 2019; Byrska-Bishop et al. 2022). The regions are particularly gene-rich, and we identify 420 protein-coding genes in 165 of them (Supplemental Table S8).

Large-scale CNVs within an assembled contig may also lead to alignment discontinuities in which contig alignment ends map far away from each other, thus exceeding the expected contig length. We identified 1721 contigs whose alignments have exceeded the absolute contig length by >5% (Fig. 1A, i). Although the majority of such contigs were observed in satellite DNA, we identified 391 contigs mapping outside of centromeric satellites, of which ∼98% are associated with SDs (Fig. 5E; Supplemental Fig. S24). Although we cannot exclude the possibility that such unusual patterns of homology result from assembly error or the inability of mapping algorithms (such as minimap2) to distinguish between paralogous sequences owing to high sequence identity (e.g., *SMN1/2* region) (Supplemental Fig. S25), complete haplotype sequence and assembly of these regions is likely to provide new insights into patterns of human genetic variation and the mutational processes that shape them (Vollger et al. 2023).

## Discussion

The recently released gapless assembly of the first haploid human genome has set the bar for T2T human genome assemblies (Nurk et al. 2022). Extending this to diploid samples requires a detailed analysis of the remaining gaps to guide new developments in both sequencing technology and assembly algorithms. Using multiple metrics, we provide a genome-wide assessment to characterize the nature of these last gaps of the human genome. There are several important conclusions. First, we show that recent improvements in sequencing technology (CLR vs. CCS) and assembly algorithms (Peregrine vs. hifiasm) reduce the number of gaps by approximately threefold. Second, the use of parental Illumina WGS data improves phased genome assembly, but the use of linked-read data such as Strand-seq or newer versions of hifiasm that incorporate Hi-C data (Cheng et al. 2021), which is much more widely available than Strand-seq, can create phased assemblies with comparable low levels of switch error. Nevertheless, both trio-based and trio-free assemblies fail to correctly resolve the orientation of 6–7 Mbp of DNA. This is especially the case for large inversion polymorphisms that are flanked by high-identity SDs, which represent one of the most difficult SV classes to accurately assemble (Chaisson et al. 2019; Porubsky et al. 2022). Such complex regions of the genome often coincide with morbid CNVs, where the critical region toggles from a direct to an inverted configuration as a result of recurrent NAHR events (Porubsky et al. 2022).

The current state-of-the-art human genome assembly is represented by approximately 140 gaps per haploid genome with about double the number when trio-free approaches, such as PGAS (Porubsky et al. 2021), are applied. Predictably, gaps cluster within copy number–variable repeat-rich locations corresponding to the largest and most identical repeats (including satellites and SDs) or within low-complexity regions enriched in GA/AT dinucleotides. The latter results from sequence coverage dropouts particular to the HiFi data type over these low-complexity regions (Nurk et al. 2020). Notably, the degree of dropout shows some dependence on the size of the dinucleotide tracts, with the majority of assembled low-complexity regions <6 kbp (Fig. 4A). Many of these regions appear to have expanded specifically in the human–primate lineage so different regions are anticipated in other nonhuman genomes. Our analysis predicts that increasing sequence coverage from 25- to 50-fold eliminates approximately two-thirds of such gaps. Although it does not totally eliminate HiFi-based errors, it has the net effect of also increasing the final base-pair accuracy. In contrast, increasing sequence coverage seems to have little effect on gaps associated with CNV SDs (Fig. 3). This is likely a consequence of the fact that insert size and sequence coverage are inversely correlated, and as a result, high-coverage samples suffer from smaller inserts that fail to resolve large SDs. In this regard, it is interesting that alternate long-read sequencing platforms, such as ONT, do not show the same inherent coverage biases toward GA/AT low-complexity repeats (Nurk et al. 2022). We estimate that, coupled with their much longer read lengths (>50 kbp), ∼64% of the remaining gaps within HiFi assemblies can be traversed by ONT (Methods) (Supplemental Fig. S26). Approaches and assembly algorithms that couple both ONT and HiFi data (e.g., Verkko) (Rautiainen et al. 2023) show considerable promise in closing the remaining gaps necessary to achieve routine T2T assemblies of human genomes. The costs of generating deep long-read sequence coverage from two platforms to generate T2T human genomes are, to date, still prohibitively high (more than $10,000), although recently announced increases in throughput from PacBio may reduce this by more than a factor of four.

One of the largest gains from T2T assemblies will be an improved understanding of human structural genetic diversity. Although still incomplete, our analysis identifies ∼6.6% and 0.06% of unaligned bases per haploid assembly localized within and outside of centromeric satellite DNA, respectively. Among such gaps caused by contig alignment discontinuities, we identify 230 regions that occurred in at least five haploid assemblies. Nearly half of these (∼40%) map to SDs where variation and incomplete assembly pose particular challenges to alignment as well as interpretation. For example, within euchromatic regions, we identified ∼15.4 Mbp of sequence per haplotype with two or more mappings per haplotype. Based on Illumina read-depth analysis, we estimate that 86% of these additional alignments represent bona fide human copy number variation. Nevertheless, ∼1.6 Mbp of the reported extra alignments are likely false as there is no support in short-read data. Of note, such alignments are often represented by contigs embedded within other larger contigs where one of the overlapping contig alignments has lost allelic variation and now carries, instead, the allelic pattern of variation of the opposing parental haplotype. Allelic variation is, however, still present but maps to only one of the contigs (mostly the shorter one) generated by trio-hifiasm for a given haplotype. This is important because current variant-calling algorithms, such as dipcall or PAV, tend to pick the longer, more contiguous contig in both haploid assemblies to infer allelic variation. We predict that such artifacts may overestimate the amount of loss-of-heterozygosity regions when the longer contig devoid of SNVs is preferentially used. These artifacts also argue that application of state-of-the art methods still requires careful curation and clean-up before their release as new references. It emphasizes the importance of assembly validation using orthogonal data sets such as short reads, optical mapping technology, or Strand-seq to flag remaining errors.

A major challenge going forward will be not only to fully sequence resolve these regions but also to represent complex SVs in such a way that they can be reliably interpreted and assayed in human genetic studies. One of the main objectives of the HPRC efforts is to project all human genome variation through a graph-based representation in which every human haplotype represents a path in the graph. Unfortunately, there are regions in current genome assemblies that are still completely missing or incorrectly assembled or that otherwise pose challenges for the construction of such pangenome graphs. A set of regions, termed “brnn” regions, were identified and “trimmed” during the construction of the minigraph-cactus graph (Liao et al. 2023). These regions were excluded at least once but, in some instances, up to 88 times and mapped predictably to satellite DNA (∼149.7 Mbp), acrocentric (∼28.9 Mbp), and SD (∼65.7 Mbp) regions and also contain protein-coding genes (n = 171) as well as common inversion polymorphisms (n = 49) (Supplemental Figs. S27–S30; Supplemental Table S9; Supplemental Notes). Here, the challenge will be not only to finish these regions but also to represent changes in meaningful ways such that ectopic exchange events among acrocentric short arms (Guarracino et al. 2023), interlocus gene conversion among SDs (Vollger et al. 2023), hypermutability, and saltatory amplifications in satellite DNA (Logsdon et al. 2021; Altemose et al. 2022) can be adequately captured. Alternate graph-based approaches, such as PGGB (Garrison et al. 2023), hold tremendous promise in this regard, but true representation of such diversity requires a fundamental understanding of the mutational processes that have shaped these regions. Therefore, teasing apart the inheritance status of complex structural variants at the familial level (Noyes et al. 2022) and a better understanding and characterization of the rate of mutational processes such as interlocus gene conversion, recurrent mutation, and duplicative transpositions based on both pedigree and population-level analyses are key (Porubsky et al. 2022; Vollger et al. 2023). Such an understanding will facilitate the development of mutation-aware alignment tools and pangenome graphs in the future.

## Methods

### Set of evaluated de novo assemblies

De novo assemblies evaluated in this study were obtained from two different sources as part of two international consortia: HGSVC and HPRC. For HGSVC data, we evaluated a panel of 35 samples of diverse ancestry (AFR, 11; AMR, 5; EUR, 7; EAS, 7; SAS, 5). Of those, there are 30 and 14 samples with PacBio CLR and CSS data, respectively (nine samples, or three trios, have both CLR and CCS data). In the HPRC assembly collection, there are 47 samples of mostly African and American ancestry (AFR, 24; AMR, 16; EUR, 1; EAS, 5; SAS, 1) sequenced using PacBio CCS data only. Of those there are five samples also assembled by HGSVC (HG00733, NA19240, HG02818, HG03486, and NA24385/HG002). This accounts for a total of 77 unique samples (35 from HGSVC and 42 from HPRC). We note that the PGAS assembly pipeline at the final step splits long-read (CLR or CCS) data into two haplotype-specific sets that are then assembled separately into haplotype-resolved assemblies (Porubsky et al. 2021).

### Alignment of de novo assemblies to the reference genome

#### Alignments used for simple contig end evaluation

All de novo assemblies were aligned to the most complete version of the human reference genome T2T-CHM13 (v1.1) using minimap2 (v2.22.0; Li et al. 2018) with the following command:

minimap2 -K 8G -t {threads} -ax asm20 \

‐‐secondary=no ‐‐eqx -s 25000 \

{input.ref} {input.query} \

| samtools view -F 4 -b - > {output.bam}

We note that minimap2 had a known issue in which some inversions were missed if they were part of another alignment. To alleviate this issue, we realigned the assemblies with the same parameters after hard masking the reference and query sequence to remove regions that were already aligned in the first alignment step. A complete pipeline for this reference alignment is available at GitHub (https://github.com/mrvollger/asm-to-reference-alignment).

The T2T-CHM13 (v1.1) reference assembly can be found at the NCBI Genome database (https://www.ncbi.nlm.nih.gov/data-hub/genome/) under accession number GCA_009914755.3.

#### Alignments used for contig alignment end evaluation

All de novo assemblies were aligned to the most complete version of the human reference genome T2T-CHM13 (v1.1) using a newer minimap2 version (v2.24.0) with the following command:

minimap2 -K 8G -t {threads} -x asm20 \

‐‐secondary=no ‐‐eqx -s 25000 \

{input.ref} {input.query} \

| samtools view -F 4 -b - > {output.bam}

A complete pipeline for this reference alignment is available at GitHub (https://github.com/mrvollger/asm-to-reference-alignment).

### Evaluation of simple contig ends

Contig ends are defined at the first and last aligned base for each contig in the HPRC haplotype-phased assemblies. Alignments were performed as described above, and the terminal position of each contig was determined using rustybam liftOver (https://github.com/mrvollger/rustybam). A complete pipeline for identifying contigs ends is included at GitHub (https://github.com/mrvollger/asm-to-reference-alignment).

### Reading in minimap2 alignments

All minimap2 alignments reported in PAF format were loaded in a set of genomic ranges using custom R (R Core Team) function “paf2ranges” with following given parameters: min.mapq = 10, min.aln.width = 1000, min.ctg.size = 100,000, report.ctg.ends = TRUE, min.ctg.ends = 50,000. At this step, we kept alignments with mapping quality equal to or more than 10 and of minimal size, 1 kbp. Also, contigs with a total size <100 kbp were filtered out.

#### Evaluation of contig alignment ends

After loading all minimap2 alignments, we extracted terminal contig alignments of at least 50 kbp. When a total alignment size of a contig to the reference was >5% of an actual contig size, we split such contigs into more than one alignment with its own alignment ends. Such splits occur in situations in which the end of the contig maps to distal SD pairs or maps across the centromere, thus increasing the mapped contig size with respect to real contig size.

#### Defining genomic regions between contig ends and discontinuities within each contig

With minimap2 alignments loaded in a set of genomic ranges, we set out to determine genomic regions spanning between them. For this, we used a custom R function (“reportGaps”) in order to report genomic ranges between subsequent contig end mappings.

### Strand-seq data generation and data processing

Strand-seq data for eight human samples (HG01123, HG01258, HG01358, HG01361, HG01891, HG02257, HG02486, and HG02559) were generated as follows. EBV-transformed lymphoblastoid cell lines from the 1 KG (1000 Genomes Project Consortium 2015) (Coriell Institute) were cultured in BrdU (100 µM final concentration; Sigma-Aldrich B9285) for 18 or 24 h, and single isolated nuclei (0.1% NP-40 substitute lysis buffer) (Sanders et al. 2017) were sorted into 96-well plates using the BD FACSMelody cell sorter. In each sorted plate, 94 single cells plus one 100-cell positive control and one zero-cell negative control were deposited. Strand-specific DNA sequencing libraries were generated using the previously described Strand-seq protocol (Falconer et al. 2012; Sanders et al. 2017) and automated on the Beckman Coulter Biomek FX P liquid handling robotic system (Sanders et al. 2020). Following 15 rounds of PCR amplification, 288 individually barcoded libraries (amounting to three 96-well plates) were pooled for sequencing on the Illumina NextSeq 500 platform (MID-mode, 75-bp paired-end protocol).

The demultiplexed FASTQ files were aligned to the T2T-CHM13 (v1.1) reference assembly using BWA aligner (v0.7.17-r1188) (Li and Durbin 2010) and SAMtools (v1.10) (Li et al. 2009). Duplicate reads were marked using sambamba (v1.0) (Tarasov et al. 2015). Low-quality libraries were excluded from future analyses if they showed low read counts, uneven coverage, or an excess of “background reads” yielding noisy single-cell data, as previously described (Porubský et al. 2016; Sanders et al. 2017). Aligned BAM files were used for assembly evaluations as described below.

### Evaluation of assembly quality using Strand-seq

For a set of eight HPRC samples (HG01123, HG01258, HG01358, HG01361, HG01891, HG02257, HG02486, HG02559) for which corresponding Strand-seq data are available, we evaluated the directional and structural contiguity of such assemblies.

#### Evaluation of misorientations and unresolved homozygous inversions

To evaluate any changes in orientation, we first processed each selected Strand-seq library using breakpointR with the following parameters: windowsize = 2,000,000, binMethod = “size,” pairedEndReads = TRUE, min.mapq = 10, genoT = “binom,” background = 0.1, minReads = 100. Next, we created so-called composite files that concatenate directional reads across all libraries using breakpointR function “synchronizeReadDir.” We set to detect any changes in directionality by running breakpointR on such composite files with the following parameters: windowsize = 10,000, binMethod = “size,” pairedEndReads = FALSE, genoT = “binom,” background = 0.1, peakTh = 0.25, minReads = 50. Misorientation and unresolved homozygous inversions are reported as regions with the majority of reads mapped in minus orientation (“ww,” Watson–Watson strand state), whereas one would expect all Strand-seq reads to map in plus orientation (“cc,” Crick–Crick strand state) if the assembly is correctly oriented throughout each contig.

#### Evaluation of phasing accuracy for selected PGAS assemblies

We evaluated phasing accuracy for HPRC samples (HG01123, HG01258, HG01358, HG01361, HG01891, HG02257, HG02486, HG02559) for which corresponding Strand-seq data are available, and thus, both HPRC and PGAS assemblies could be produced. In this analysis, we consider trio-based HPRC assemblies as the gold standard for phasing evaluation. We used PAV (v1.1.2) to call SNVs in phased HPRC assemblies as described previously (Ebert et al. 2021). To search for large-scale switch errors, we split phased PGAS assemblies into 1-Mbp-long chunks. Subsequently, we used WhatsHap (v1.0) (Patterson et al. 2015) to assign each 1-Mbp chunk to either haplotype 1 or 2 based on a trio-based set of phased SNVs. For each sample, we evaluated a fraction of wrongly assigned 1-Mbp segments separately for haplotype 1 and 2 across all autosomes. Visually, we detected two large-scale switch errors on Chromosome 9 in sample HG01891. There was one switch error around position 42 Mbp (near the centromere); the other, near the end of Chromosome 9 at position 137.3 Mbp.

#### Evaluation of inversion resolution for selected PGAS assemblies

To evaluate the performance of trio-based and trio-free assemblies to resolve inversion, we selected a set of large inversions (≥100 kbp) from the previous study (Porubsky et al. 2022). We mapped inversion coordinates from GRCh38 to T2T-CHM13 (v1.1) coordinates using minimap2 (v2.20) using following parameters: ‐‐secondary=no ‐‐eqx -ax asm20 -r 100,1k -z 10000,50. We selected a set of 20 inverted sites (≥100 kbp) with a clear Strand-seq inversion pattern. For dotplot visualization purposes, we added extra padding on each side of the inversion equal to the size of the inversion or minimum of 2 Mbp. We extracted assembly alignments to the reference T2T-CHM13 (v1.1) corresponding to these regions from each trio-based and trio-free phased assembly using rustybam (v0.1.27) function “liftover.” Next, we exported a FASTA file from each assembly based on subsetted region-specific PAF files. We used NUCmer (MUMmer v3.23; Delcher et al. 2002) with the parameters ‐‐mum ‐‐coords to align each FASTA file to the reference sequence (T2T-CHM13 v1.1). We visualized alignments for each assembly in each inverted region as dotplot. Each dotplot was evaluated manually. Inversion was deemed to be resolved if an inversion can be traced in a single contig in both haplotypes and if the inversion status in both haplotypes matches the reported inversion genotype presented by Porubsky et al. (2022).

### Definition of centromeric satellite DNA

In this study, centromeric satellite DNA was defined based on T2T-CHM13 annotation obtained from UCSC Table Browser. Annotation was obtained for T2T-CHM13 (v1.1) reference from the annotation group “centromeres and telomeres” and annotation track “CenSat annotation.” We define centromeric satellite DNA as regions annotated as human-satellites (hsat), beta-satellites (bsat), and alpha-satellites HOR array (hor).

### Protein-coding gene annotations

Gene annotation used in this study is based on T2T-CHM13 annotation obtained from UCSC Table Browser. Annotation was obtained for T2T-CHM13 (v1.1) reference from the annotation group “genes” and annotation track “CAT genes+LiftOff V4.” When reporting gene overlap, we selected only protein-coding genes. Any T2T-CHM13-specific genes were not considered. Lastly, subsequent ranges of the same gene were collapsed.

### Evaluation of ONT alignments

Available ONT reads (obtained from the NCBI BioProject database [https://www.ncbi.nlm.nih.gov/bioproject/] under accession number PRJNA731524) for 33 HPRC samples were aligned to the T2T-CHM13 (v1.1) reference assembly using minimap2 (v2.24) and filtered secondary alignments using SAMtools (v1.9). We ran the alignments with the following parameters:

minimap2 -a -t {threads} -I 10G -Y -x map-ont {assembly} {fastq} | samtools view -u -F 256 - | samtools sort -o {bam_name} -

Obtained alignments were exported as read alignment positions in BED format. Only reads with mapping quality 10 or greater were retained for further analysis. We tested each reported assembly gap region per sample and per haplotype if such a region is spanned by 10 or more ONT reads to assume that such assembly gap could eventually be closed by underlying ONT reads. The download locations for ONT data are also reported in Supplemental Table S10.

### Low-complexity regions among frequent assembly breaks

Out of the total 592 defined frequent assembly breaks, we extracted 44 regions where there is an assembly break in half or more of the HPRC assemblies. Next, we extracted the T2T-CHM13 FASTA sequence corresponding to these regions (n = 44). We calculated the total number of three dinucleotides (TA, TC, and GA) in nonoverlapping 100-bp-long sequence chunks (bins). To define dinucleotide-enriched bins, we transformed binned dinucleotide counts into the *Z*-scores and marked bins with *Z*-score ≥1.96 (95% confidence interval) as dinucleotide enriched. The size of dinucleotide tracts was estimated as the number of enriched bins × 100 (bin size).

We also investigated FASTA sequence from the previously defined regions (n = 44) in all HPRC assemblies along with nonhuman primate assemblies (n = 18). We processed only those assemblies that span defined regions in a single contig and map to defined breakpoints in T2T-CHM13 coordinates (±100 bp). Next, we transformed observed dinucleotide counts into *Z*-scores as outlined above. Based on visual inspection, we selected 27/44 regions with observable differences in the size of dinucleotide tracts between human and nonhuman primate assemblies (Supplemental Table S6).

### Defining regions of putative structural variation

We examined large contig alignment discontinuities as gaps within a single contig alignment that are <1 Mbp. We classified a contig alignment discontinuity as a “contraction” if the alignment gap (in target sequence coordinates) is larger than the corresponding gap within a contig (in query sequence coordinates) (Fig. 1A, iii). In contrast, we classified a contig alignment discontinuity as an “expansion” if the alignment gap (in target sequence coordinates) is smaller than the corresponding gap within a contig (in query sequence coordinates). The number of unaligned bases is defined as the size of the gap in query sequence coordinates. The predicted size of the contractions and expansions was defined as a difference in size between gap in target and query coordinates. We marked contig alignment discontinuities that are within or close (±1 Mbp) to centromeric satellite DNA (marked as “CENSAT”) because contig assemblies and alignments within and near centromeres are complicated by the repetitive nature of centromeric satellites and high degree of SDs in these regions. We summarized predicted sites of contraction and expansion into a set of nonredundant regions constructed from sites where contraction and expansion are observed in at least five assemblies and the predicted event size is ≥100 bp (Supplemental Table S7).

### Detection of CNV regions

To define regions that are likely copy number variable in any given sample, we searched for regions where there are overlapping contig alignments with respect to the T2T-CHM13 (v1.1) reference. In this analysis, we considered only autosomes, and we filtered out regions that overlap centromeric satellites. We opt to validate putative CNV regions using short-read-based copy number profiles obtained for 44/47 HPRC samples. Short Illumina reads were computationally parsed into 36-bp segments and aligned to a hardmasked T2T-CHM13 (v1.1) reference using mrsFAST (Hach et al. 2010), allowing an edit distance of two. Read-depth-based copy number estimates were generated using the FastCN (Pendleton et al. 2018) software package, which uses known copy number stable regions to correct for Illumina sequencing GC bias and convert read depth to diploid copy number over windows containing 500 unmasked base pairs.

Because of the mapping of short reads to a single paralogous copy in the genome, we set out to determine sample-specific copy number by establishing reference copy number of paralogous regions in T2T-CHM13 (v1.1). We did this by splitting T2T-CHM13 (v1.1) sequence into the same 36-bp subsequences with a slide of one to cover all *k*-mers in the reference. These *k*-mers were mapped back to the reference using mrsFAST, and copy number was determined via FastCN. We refer to this as the *k*-mer-ized T2T-CHM13 reference copy number.

We defined sample-specific CNV regions as those with a diploid copy number less than 10 and at least one diploid copy number increase compared with the *k*-mer-ized T2T-CHM13 reference copy number. Sample-specific regions with a diploid copy number of two and/or no difference (delta = 0) from the *k*-mer-ized T2T-CHM13 reference copy number were defined as not copy number variable and marked as “noCN.” Regions where there is an observable diploid copy number increase yet the overall sample-specific copy number is greater than 10 were marked as “more10CN.” Regions that do not fall into any of the above categories were marked as “none.”

### Analysis of pangenome brnn regions

Genomic regions that were excluded from the T2T-CHM13-based pangenome graph construction were obtained from GitHub (https://github.com/human-pangenomics/hpp_pangenome_resources#masked-sequenc). A detailed description of how these regions were defined is reported in the link above. We next took the file “hprc-v1.0-mc-chm13.clipped-intervals.bed.gz,” and for each genomic region, we extracted the FASTA sequence from a corresponding phased assembly. We then aligned these to the T2T-CHM13 (v1.1) reference using minimap2 (v2.24) with the following parameters: ‐‐secondary=no ‐‐eqx -ax asm20 -r 100,1k -z 10000,50. Finally, we kept only alignments of minimum mapping quality of 10 or more and also excluded any alignments from mitochondrial DNA.

### Generation of DeepVariant single-nucleotide polymorphism calls for false loss of heterozygosity detection

Alignments of raw PacBio HiFi reads (from seven samples: HG02486, HG02572, HG02622, HG02886, HG03516, HG03540, HG03579) to T2T-CHM13 (v1.1) were made with pbmm2 (https://github.com/PacificBiosciences/pbmm2) using the “CCS” preset. DeepVariant calls were generated using DeepVariant (v1.4.0) (Poplin et al. 2018) and the “PACBIO” pretrained model. PacBio HiFi reads are available at the NCBI BioProject database under the accession number PRJNA731524.

## Data access

All raw and processed sequencing data generated in this study have been submitted to the NCBI BioProject database (https://www.ncbi.nlm.nih.gov/bioproject/) under accession number PRJEB54100. The DeepVariant callsets for selected samples (HG02486, HG02572, HG02622, HG02886, HG03516, HG03540, HG03579) and FASTA sequences from selected low-complexity regions (n = 27) are available at Zenodo (https://doi.org/10.5281/zenodo.7392259) or at the IGSR FTP site (http://ftp.1000genomes.ebi.ac.uk/vol1/ftp/data_collections/HGSVC2/working/publications/202212_Porubsky_GenomeResearch). All custom scripts are available in the Supplemental Code and at Zenodo (https://doi.org/10.5281/zenodo.7392259).

## Supplementary Material

Supplemental Material
