# Supplementary material for: Gaps and complex structurally variant loci in phased genome assemblies
Source: Genome Res. 2023 Apr;33(4):496–510. doi: 10.1101/gr.277334.122 (PMC10234299; doi:10.1101/gr.277334.122)
Supplement: Supplemental Material [file supp_33_4_496__DC1.html]

Gaps and complex structurally variant loci in phased genome assemblies — Gaps and complex structurally variant loci in phased genome assemblies — Supplemental Material 

# Gaps and complex structurally variant loci in phased genome assemblies

## Supplemental Material

- Supplemental\_Materials.pdf
- Supplemental\_Tables.xlsx
- Supplemental\_Code.zip
